# Aortic Dissection Complicated by Mesenteric Malperfusion Syndrome Presenting As Hepatic Ischemia: A Case Report and Literature Review

**DOI:** 10.7759/cureus.74139

**Published:** 2024-11-21

**Authors:** Bashir Mahamud, Shoayeb Sarwar, Lina Eltayieb, Hussameldin Mahdi, Gideon Mlawa

**Affiliations:** 1 Acute Medicine, Barking, Havering and Redbridge National Health Service (NHS) Hospital Trust, London, GBR; 2 Internal Medicine, Barking, Havering and Redbridge National Health Service (NHS) Hospital Trust, London, GBR; 3 Internal Medicine and Diabetes and Endocrinology, Barking, Havering and Redbridge National Health Service (NHS) Hospital Trust, London, GBR

**Keywords:** aortic dissection, cardiac arrest, hepatitis ischemia, malperfusion syndrome, mesenteric ischemia

## Abstract

Aortic dissection (AD) is a medical emergency that occurs as a result of a compromise in the structural integrity of the aorta. If left untreated, AD can have severe consequences such as organ dysfunction or even death. Malperfusion syndrome is a major complication of aortic dissection with mesenteric malperfusion syndrome being a rare but devastating form that can lead to mesenteric ischemia and is associated with poor prognosis despite timely management.

Here we report a case of a 31-year-old woman who was diagnosed with mesenteric ischemia secondary to incidental findings of aortic dissection whilst being investigated for ischemic hepatitis. She underwent emergency surgery to repair the aortic dissection but faced challenges due to unusual arterial vasculature. Despite best efforts, the patient's condition deteriorated, leading to severe brain injury.

Management of aortic dissection complicated by mesenteric malperfusion remains a clinical challenge with high mortality rates and despite this, there is currently no definitive national guideline for the best management approach.

## Introduction

Aortic dissection (AD) is a condition characterized by a rupture in the inner lining of the aorta. AD results in the formation of a false lumen within the aortic wall, which can extend along the length of the vessel and compromise its structural integrity. If not promptly addressed, AD may lead to severe complications, including inadequate organ perfusion, cerebrovascular events, or even fatality. The condition demands immediate intervention to prevent catastrophic outcomes [1-3].

Classically, AD presents with sequelae of chest pain radiating to the back, asymmetric upper limb blood pressure readings, and associated neurological deficit, however, it can also present with atypical features. Diagnoses of AD can be challenging, partly, due to its features mimicking many other conditions and its diagnosis requires high clinical suspicion and accurate history-taking [4-6]

Moreover, 30% of patients with aortic dissection often suffer complications from distal vascular supply leading to the so-called malperfusion syndromes. Malperfusion syndrome can affect most organ systems including the visceral/mesenteric. Mesenteric malperfusion syndrome (MMP), although rare, is a devasting form of malperfusion syndrome, that can lead to mesenteric ischemia. MMPs secondary to aortic dissection have poor prognosis and high mortality rates despite modern intervention [7-8].

Herein we present a case of a patient who was diagnosed with mesenteric ischemia secondary to incidental findings of aortic dissection following investigation to rule out ischemic hepatitis.

## Case presentation

**Figure 1:**
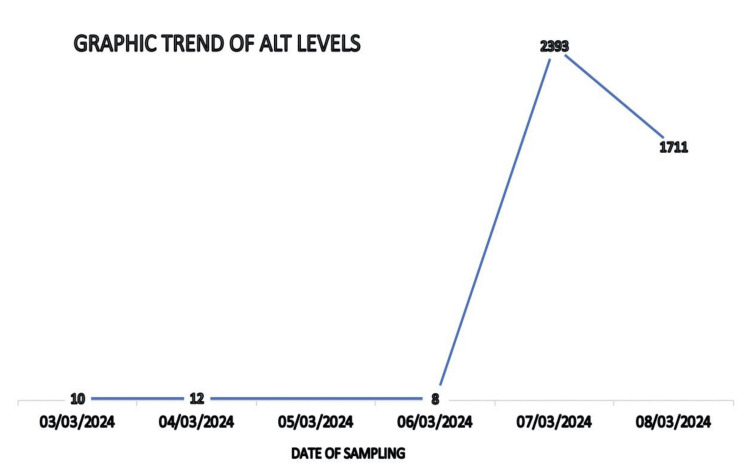
Graphic trend of patient's alanine transferase (ALT) levels.

A 31-year-old woman presented to our trust complaining of persistent vomiting and diarrhea, which prompted her to seek medical attention. Blood tests showed that the patient had acute hepatic dysfunction with high aspartate transaminase (AST) and alanine transaminase (ALT) levels (Table 1, Figure 1).

**Table 1:** Biochemistry results illustrating patients deranged liver function test. eGFR: estimated glomerular filtration rate, AST: aspartate transaminase, GGT: gamma-glutamyl transferase.

	07/03/2024	08/03/2024	Reference range
Sodium	135	128	133-146 mmol/L
Potassium	4.3	4.4	3.5-5.3 mmol/L
Urea	22	29.1	2.5-7.8 mmol/L
Creatinine	511	616	45-84 umol/L
eGFR	9	7	>90 mL/min
Alkaline phosphatase	113	106	30-130 U/L
Alanine transferase	2393	1711	<33 U/L
AST	2379	948	<32 U/L
Total bilirubin	15	12	1-21 umol/L
Calcium	2.53	2.59	2.20-2.60 mmol/L
GGT	31	33	<40 U/L

**Figure 2:**
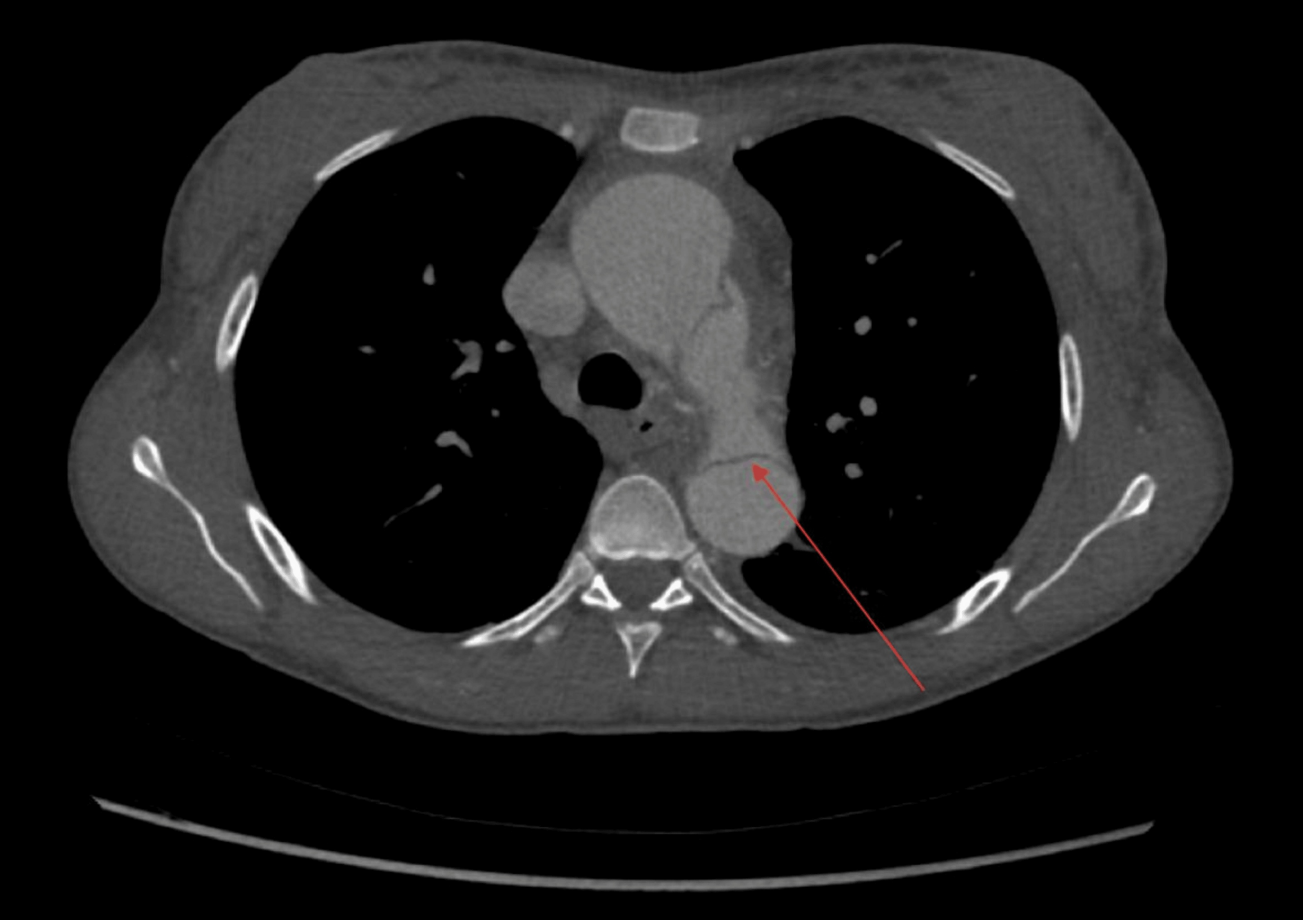
CT scan highlighting dissection flap, also visible is small true lumen and larger false lumen.

As a result, she underwent further investigation to rule out ischemic hepatitis including a computed tomography (CT) scan, which revealed subacute type A aortic dissection with a small right lateral true lumen and a larger left lateral false lumen. The false lumen expanded into the coeliac axis, superior mesenteric artery (SMA), and left renal artery. The aortic dissection was complicated by a thrombus extending into the coeliac axis (Figure 2). The patient started to deteriorate and became hemodynamically unstable.

Recognizing the urgency of the situation, the patient was transferred to a tertiary center for emergent surgery to repair the aortic dissection. However, surgery was complicated by challenging peripheral access, as a result of unusual arterial vasculature. The patient also suffered cardiac arrest requiring seven minutes of internal massage and emergent bypass initiation.

Post-surgery patients' hepatic function did not improve, and her lactate level began to rise. She later suffered a drop in her consciousness level and CT Head showed global cerebral edema in keeping with severe global hypoxic-ischaemic brain injury and bilateral cerebellar infarcts. 

## Discussion

Acute AD is a life-threatening condition with high mortality and morbidity rates. Various risk factors, such as hypertension, genetic predisposition, connective tissue disorders, atherosclerosis, and trauma, have been linked to the development of AD. AD is categorized according to the Stanford classification as either type A dissection which involves the ascending aorta, and type B dissection which does not [9-11]. 

Acute AD presents with varying manifestations, and classic textbook features are often absent, thus in order to make an accurate diagnosis, a high clinical index of suspicion is required. AD can lead to multiple complications including the so-called malperfusion syndrome which is associated with significantly high mortality rates. Malperfusion syndrome complicates up to 35% of AD owing to the anterograde spread of dissection from the ascending aorta to the level of the aortic visceral branches which can impact any vascular bed with varying degrees of effect on the end-organ [6,7,12-13]. 

The case reported here details a patient who presented with nausea and vomiting, and blood tests done during admission demonstrated acute derangement liver function (Table 1) with significant elevated ALT (Figure 1). This led to the patient being investigated for ischemic hepatitis and she subsequently underwent a CT scan.

Type A AD with thrombus in the coeliac trunk and its branches, proximal inferior mesenteric artery, and left renal artery was found on a CT scan (Figure 2) and later CT angiogram reported findings in keeping with mesenteric malperfusion and splenic infarct. Despite the patient having urgent surgical treatment, the patient continued to deteriorate. Despite the best efforts she tragically succumbed to her condition and passed away.

Type A AD even without any associated complication remains a surgical emergency. However, AD complicated by MMPs is a clinical challenge, with a high mortality rate, and is associated with an increased risk of mesenteric ischemia. A recent study by the International Registry of Acute Aortic Dissection highlighted that MMPs are the second leading cause of death in patients with AD after aortic rupture [7]. AD with MMPs has an insidious onset and lack of definitive diagnostic classification contributes to delay in referral to tertiary hospitals. Remarkably, up to 40% of the patients with MMPs do not develop characteristic features such as abdominal pain seen in bowel ischemia. It is believed that the poor outcome associated with Mesenteric malperfusion has been attributed to the fact that it causes an inflammatory cascade that leads to end-organ injury, which can persist even after aortic repair [13-14]. 

With MMPs timely management plays a crucial role in determining favorable outcomes for patients. Currently, there are no definitive national guidelines for the management of AD complicated by MMPs. Surgical intervention remains the gold standard treatment, however, surgeons are conflicted with the decision on which lesion should be treated first the aorta or the visceral, and how the compromised arteries are to be reperfused. The traditional strategy for the management of AD with MMPs has been central aortic repair, although this restores true lumen flow, branch vessel ischemia can persist due to the presence of distal re-entry tears and persistent false lumen flow. In a recent review, it was found that in-hospital mortality following central aortic repair as the main treatment strategy was greater than reperfusion of SMA as the first line of management [13-15].

Recent studies have advocated that for patients with acute type A AD with MMPs with rising serum lactate levels and bowel ischemia correcting MMPs is associated with favorable short- and long-term outcomes. Endo-vascular fenestration/stenting with delayed open aortic repair can resolve visceral malperfusion quickly and adequately and improve the chance of recovery from critical organ failure and survival with subsequent open aortic repair [14-17].

Recently, Ni et al. reported on the use of a one-stop hybrid approach to manage patients with AD complicated by malperfusion of the superior mesenteric artery [18]. In a hybrid operation room, the patient initially underwent endovascular bare-metal stenting of the superior mesenteric artery which successfully restored visceral perfusion. Then aortic root repair with debranching total arch replacement and retrograde stent graft implantation was performed. The strengths of this technique are that it first relieves visceral malperfusion and prevents circulatory arrest due to arch repair, through debranching the supra-aortic vessels. Subsequent CT scans post-surgically and before patient discharge showed patent visceral vessels [18-19].

## Conclusions

Management of AD complicated by visceral malperfusion requires a multi-disciplinary, patient-centered approach. Mesenteric ischemia resulting from AD remains a diagnostic challenge for clinicians, which often leads to delays in management. Worryingly, there are no definitive national guidelines to help clinicians decide the management approach. We hope that this case report will raise clinical suspicion and prompt early investigation and management. We also believe that national guidelines advocating for definitive management options would help with decision-making regarding intervention type and enable timely decision making which would have favorable patient outcomes.
